# Beyond sicca: high prevalence and predictors of baseline and worsening systemic involvement in patients with Sjögren’s disease

**DOI:** 10.1093/rap/rkae035

**Published:** 2024-03-06

**Authors:** Matilde Bandeira, Manuel Silvério-António, Nikita Khmelinskii, João E Fonseca, Vasco C Romão

**Affiliations:** Rheumatology Department, Unidade Local de Saúde Santa Maria, Centro Académico de Medicina de Lisboa, Lisboa, Portugal; Rheumatology Research Unit, Instituto de Medicina Molecular, Faculdade de Medicina, Universidade de Lisboa, Centro Académico de Medicina de Lisboa, Lisboa, Portugal; Rheumatology Department, Unidade Local de Saúde Santa Maria, Centro Académico de Medicina de Lisboa, Lisboa, Portugal; Rheumatology Research Unit, Instituto de Medicina Molecular, Faculdade de Medicina, Universidade de Lisboa, Centro Académico de Medicina de Lisboa, Lisboa, Portugal; Rheumatology Department, Unidade Local de Saúde Santa Maria, Centro Académico de Medicina de Lisboa, Lisboa, Portugal; Rheumatology Research Unit, Instituto de Medicina Molecular, Faculdade de Medicina, Universidade de Lisboa, Centro Académico de Medicina de Lisboa, Lisboa, Portugal; Rheumatology Department, Unidade Local de Saúde Santa Maria, Centro Académico de Medicina de Lisboa, Lisboa, Portugal; Rheumatology Research Unit, Instituto de Medicina Molecular, Faculdade de Medicina, Universidade de Lisboa, Centro Académico de Medicina de Lisboa, Lisboa, Portugal; Rheumatology Department, Unidade Local de Saúde Santa Maria, Centro Académico de Medicina de Lisboa, Lisboa, Portugal; Rheumatology Research Unit, Instituto de Medicina Molecular, Faculdade de Medicina, Universidade de Lisboa, Centro Académico de Medicina de Lisboa, Lisboa, Portugal

**Keywords:** Sjögren’s syndrome, risk factors, predictors, ESSDAI, systemic involvement

## Abstract

**Objectives:**

Systemic extraglandular involvement in SS has been reported in one-third of patients but may be more frequent. We aimed to evaluate systemic disease prevalence at baseline and throughout follow-up and find its predictors.

**Methods:**

We conducted a retrospective cohort study including SS patients followed in a tertiary centre. The cumulative EULAR SS disease activity index (ESSDAI) was calculated by adding each domain’s maximum score throughout follow-up. We identified independent predictors of systemic involvement (ESSDAI ≥1 at baseline and/or follow-up) through logistic regression modelling. A survival analysis was conducted to identify predictors of new/worsening ESSDAI domains.

**Results:**

A total of 216 patients were included, most of whom had systemic involvement (86%), frequently at diagnosis (76%). Biological (53%) and articular ESSDAI domains (44%) were most commonly involved, but all were affected at least once. Around half of the patients with baseline systemic disease developed an additional/worsening domain throughout follow-up. Although most patients had low disease activity at baseline, 60% eventually reached moderately active disease. Younger age at diagnosis [odds ratio (OR) 0.95 (95% CI 0.91, 0.99)], a positive minor salivary gland biopsy [OR 4.08 (95% CI 1.40, 11.86)] and RF [OR 4.67 (95% CI 1.52, 14.33)] were independent predictors of systemic involvement. Patients with baseline constitutional involvement [hazard ratio (HR) 2.23 (95% CI 1.13, 4.40)] and RF [HR 1.89 (95% CI 1.20, 3.00)] were more likely to develop new/worsening systemic disease activity.

**Conclusion:**

Systemic involvement is seen in most SS patients. Younger and RF and salivary gland biopsy-positive patients are at higher risk of systemic disease. Around half of patients with systemic involvement experienced aggravated disease over time, especially those with constitutional involvement or RF.

Key messages

- Systemic involvement is seen in the majority of SS patients.
- Younger age at diagnosis and positive biopsy and rheumatoid factor are predictors of systemic disease.
- Half of the patients experience systemic disease worsening, especially those with constitutional involvement or RF.

## Introduction

SS is a systemic immune-mediated rheumatic disease targeting mainly the exocrine glands. Its clinical hallmarks are ocular and oral dryness, accompanied by disabling fatigue and pain. Systemic extraglandular involvement has been classically described in one-third of patients [1]. However, there is evidence suggesting that it may be more common [2].

Stratification of SS patients is a major unmet need [3]. Predicting if and when systemic involvement will occur allows differentiation of distinct disease profiles and prognosis. Data suggest that patients with a greater systemic disease burden are at higher risk of developing lymphoma, persistent high disease activity and poor long-term outcome [4, 5]. However, there are scarce data on how systemic disease develops throughout time. This study aimed to find predictors of prevalent and/or incident systemic disease in SS.

## Methods

We conducted a retrospective cohort study including SS patients fulfilling the 2016 ACR/EULAR classification criteria and actively followed in our centre between September 2021 and March 2022. Data were retrieved from the patients’ local clinical files.

Systemic involvement was defined as a EULAR SS disease activity index (ESSDAI) ≥1, either at the time of diagnosis or at any point during follow-up. A cumulative ESSDAI was calculated, as previously proposed by Risselada *et al.* [6], by adding the maximum scores of each domain during follow-up. Patients were divided into three groups based on the ESSDAI: no systemic involvement (ESSDAI = 0 at all time points), incident systemic involvement (ESSDAI at diagnosis = 0 and ≥1 at any point of follow-up) and baseline systemic involvement (ESSDAI at diagnosis ≥ 1).

Descriptive statistics were presented as mean (s.d.) for continuous variables and frequencies for categorical variables. Univariate analysis was performed using the chi-squared, Fisher’s exact, Mann–Whitney or *t*-test, as appropriate. Predictors of systemic involvement were identified through binomial logistic regression modelling. Correlated variables and cases with missing information were excluded from the model to ensure the validity of the regression. We performed a survival analysis for new/worsening ESSDAI domains over time through a Kaplan–Meyer survival function. We explored predictors of this combined outcome using multivariate Cox regression. The statistical analysis was performed with SPSS version 23 (IBM, Armonk, NY, USA) and statistical significance was set at *P* < 0.05.

The study was conducted according to the principles of the Declaration of Helsinki as amended by the 64th World Medical Association General Assembly, Fortaleza, Brazil, October 2013. All patients signed an informed consent. Approval was obtained from the Ethics Committee of Centro Académico de Medicina de Lisboa (169/22). All data were pseudo-anonymized.

## Results

We included 216 patients fulfilling the classification criteria (out of 292 with a clinical diagnosis of SS), 96.8% of whom were female, with a mean age at diagnosis of 51.3 years (s.d. 14.9) and a mean disease duration (follow-up) of 9.0 years [s.d. 7.8; median 6.0 years (IQR 10)] (Table 1). The majority of patients had oral (94.0%) or ocular dryness (91.7%) and were positive for anti-SSA (91.7%), anti-SSB (54.4%), ANA (93.1%) or RF (52.6%). Unstimulated whole salivary flow and Schirmer’s test were reduced in 43% and 69.4% of patients, respectively. A focus score ≥1 was seen in 68.2% of patients with a mean of 2.5 (s.d. 1.5).

**Table 1.** Clinical and laboratory features of patients with SS based on the pattern of systemic involvement

Characteristics	Overall (*N* = 216)	No systemic involvement^a^ (*n* = 31)	Incident systemic involvement^b^ (*n* = 22)	Systemic involvement at baseline^c^ (*n* = 163)	Univariate analysis *P*-value	Multivariate analysis^d^, OR (95% CI), *P*-value
Age at diagnosis, years, mean (s.d.)	51.3 (14.9)	**58.2 (13.9)**	**51.3 (11.1)**	**50.0 (15.2)**	**0.029**	**0.95 (0.91, 0.99), 0.022**
Disease duration, years, mean (s.d.)^e^	9.0 (7.8)	**5.9 (5.8)**	**11.3 (9.5)**	**9.2 (7.7)**	**0.041**	1.08 (0.97, 1.20), 0.160
Female, *n* (%)	209 (96.8)	30 (96.8)	21 (95.5)	158 (96.9)	0.935	0.47 (0.04, 5.78), 0.552
Ocular dryness, *n* (%)	198 (91.7)	29 (93.5)	21 (95.5)	148 (90.8)	0.698	0.47 (0.06, 3.87), 0.486
Oral dryness, *n* (%)	203 (94.0)	30 (96.8)	21 (95.5)	152 (93.3)	0.717	1.21 (0.09, 11.86), 0.885
Schirmer’s test <5 mm/5 min, *n* (%)	129 (69.4)	19/26 (73.1)	13/18 (72.2)	97/142 (68.3)	0.856	
Unstimulated salivary flow <0.1 ml/min, *n*(%)	52 (43.0)	5/17 (29.4)	3/11 (27.3)	44/93 (47.3)	0.213	
Positive MSGB^f^, n (%)	122 (68.2)	**12/27 (44.4)**	**15/19 (78.9)**	**95/133 (71.4)**	**0.013**	**4.08 (1.40, 11.86), 0.010**
Focus score, mean (s.d.)	2.5 (1.5)	2.0 (1.2) (8)	2.6 (1.7) (9)	2.6 (1.5) (57)	0.414	
Anti-SSA/Ro, *n* (%)	198 (91.7)	27 (87.1)	21 (95.5)	150 (92.0)	0.525	2.89 (0.58, 14.44), 0.196
Anti-SSB/La, *n* (%)	117 (54.4)	16 (51.6)	13 (61.9)	88 (54.0)	0.746	0.51 (0.16, 1.60), 0.249
ANA, *n* (%)	201 (93.1)	27 (87.1)	20 (90.9)	154 (94.5)	0.306	
RF, *n* (%)	110 (52.6)	**7/28 (25.0)**	**11/20 (55.0)**	**92/161 (57.1)**	**0.007**	**4.67 (1.52, 14.33), 0.007**
Haematologic neoplasia, *n* (%)	11 (5.1)	0 (0.0)	1 (4.5)	10 (6.1)	0.360	
Cumulative ESSDAI, mean (s.d.)	8.4 (8.1)	0 (0)	7.6 (7.4)	10.0 (7.9)	0.097^g^	
ESSDAI domains involved, mean (s.d.)	2.3 (1.8)	0 (0)	**1.9 (1.1)**	**2.8 (1.6)**	**0.008** ^g^	

Bold indicates *P* < 0.05.

a Patients with ESSDAI = 0 at diagnosis and throughout follow-up.

b Patients that developed systemic involvement at follow-up (ESSDAI at diagnosis = 0 and ESSDAI throughout follow-up ≥1).

c Patients with systemic involvement at baseline (ESSDAI at diagnosis ≥1).

d For the prediction of baseline/incident systemic involvement *vs* no involvement.

e Equivalent to time of follow-up.

f Defined as a focus score ≥1.

g Comparing only both groups with systemic involvement.

Systemic involvement at any point was observed in a large proportion of patients (85.6%; Supplementary Fig. S1, available at *Rheumatology Advances in Practice* online). The ESSDAI biological domain, characterized by laboratory features of B cell hyperactivity, was most frequently involved (53%), followed by articular involvement (44%). Salivary gland swelling and haematological involvement were each observed in 61 patients (28%), whereas cutaneous and constitutional involvement were seen in 51 (24%) and 47 (22%) patients, respectively. Pulmonary, renal, muscular and neurological involvement were less frequent (<10%). Of note, all ESSDAI domains were involved at least once and patients had a mean involvement of 2.3 domains [s.d. 1.8; median 2.0 (IQR 2.0)] throughout disease course. More than 80% of patients [*n* = 185 (85.6%)] had one or more domains [*n* = 47 (21.8%)] involved throughout follow-up. Two patients (0.9%) had a maximum of eight active domains overall (Supplementary Table S1, available at *Rheumatology Advances in Practice* online).

Three-quarters of patients [*n* = 163 (75.5%)] had baseline systemic involvement (Table 1), more commonly having one (*n* = 69) or two (*n* = 51) active domains, with a highest of five domains observed in seven patients. A total of 41.5% (*n* = 22/53) of patients without baseline systemic disease later developed it. In terms of new-onset disease that involved ESSDAI domains, 44.4% of patients (*n* = 96) developed at least one more domain throughout follow-up, regardless of baseline disease activity. Half of these (*n* = 48) developed only one additional domain.

Importantly, roughly half of the patients with systemic disease at diagnosis [*n* = 82/163 (50.3%)] developed either additional (*n* = 74) or aggravation of systemic involvement throughout follow-up, with the other half maintaining a stable cumulative ESSDAI. The specific domains involved at diagnosis and follow-up differed slightly (Supplementary Fig. S2, available at *Rheumatology Advances in Practice* online). Of note, the biological and articular domains were most commonly active at baseline, whereas haematological involvement was more frequently active later on.

The three patients’ phenotypes differed in several characteristics (Table 1). Patients without any systemic involvement were older, with lower disease duration and less frequently RF and minor salivary gland biopsy (MSGB) positive.

**Figure 1.**
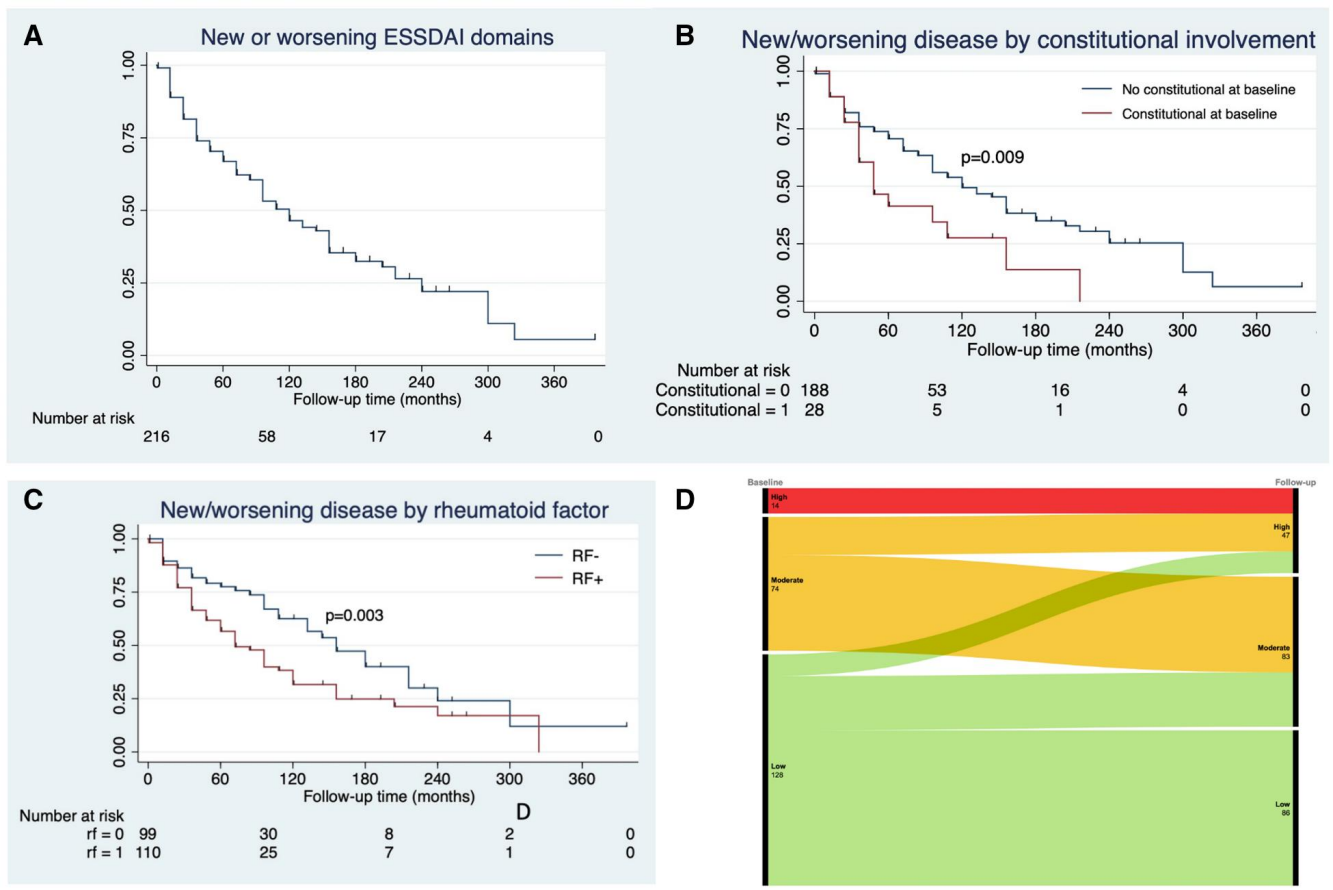
New or worsening ESSDAI domains throughout follow-up in (**A**) the entire cohort and according to (**B**) constitutional involvement at baseline and (**C**) RF positivity. (**D**) Changes in the distribution of patients with low, moderate and high ESSDAI from baseline to follow-up

The cumulative ESSDAI was higher in patients with baseline *vs* incident systemic involvement (Table 1). In addition, cumulative ESSDAI was increased in anti-SSA- [8.7 (s.d. 8.2) *vs* 4.0 (s.d. 4.2), *P* = 0.013], ANA- [8.7 (s.d. 8.2) *vs* 3.9 (s.d. 4.2), *P* = 0.017] or RF-positive [10.0 (s.d. 8.3) *vs* 6.4 (s.d. 6.9), *P* < 0.001] patients and correlated with younger age at diagnosis (*r_s_* = −0.28, *P* < 0.001) and longer disease duration (*r_s_* = 0.23, *P* = 0.001). When considering the disease activity class based on ESSDAI values (0–4, low; 5–13, moderate; ≥14, high), more than half of the patients with systemic involvement at baseline had low disease activity [128/216 (59.3%); Fig. 1D]. Throughout follow-up, the majority of patients [130/216 (60.2%)] attained at least moderate disease activity. However, a relevant group of patients maintained low disease activity [86/216 (39.8%)].

The presence of any systemic involvement (ESSDAI ≥1 at diagnosis or follow-up) was independently associated with younger age at diagnosis [odds ratio (OR) 0.95 (95% CI 0.91, 0.99)], positive MSGB [OR 4.08 (95% CI 1.40, 11.86)] and RF [OR 4.67 (95% CI 1.52, 14.33)], adjusting for sex, disease duration, anti-SSA/B status and sicca symptoms (Table 1).

The median time to disease worsening, i.e. the development of more or novel systemic activity over follow-up (new/worsening ESSDAI domains), was ≈9 years (108 months; Fig. 1A). Of note, patients with baseline constitutional involvement or who were RF positive were more likely to develop new or worsening systemic disease (Fig. 1B and C). Multivariate analysis confirmed constitutional involvement at diagnosis [hazard ratio (HR) 2.23 (95% CI 1.13, 4.40), *P* = 0.020] and RF [HR 1.89 (95% CI 1.20, 3.00), *P* = 0.007] as independent predictors of disease worsening during follow-up, irrespective of age, sex, disease duration, anti-SSA/B, MSGB and baseline disease activity (Supplementary Table S2, available at *Rheumatology Advances in Practice* online).

## Discussion

Prognostic factors in SS have been investigated, particularly markers of systemic complications of SS, as these are associated with poor long-term outcomes. Our study identified younger age, RF and a positive MSGB as independent predictors of systemic involvement.

In this study we report a substantially higher prevalence of systemic disease, defined by ESSDAI, compared with what has been traditionally acknowledged [7]. The majority of patients (86%) in our cohort did, in fact, demonstrate at least one active ESSDAI domain, with the biological and articular domains playing a predominant role. In addition, constitutional, glandular, haematological, lymphadenopathic and cutaneous involvement were each observed in ≈20–30% of patients. It is also relevant to mention that some of these previous studies [7] reported on systemic involvement as specific organ involvement and not based on the ESSDAI, which may lead to prevalence differences. Additionally, there are reports of involvement not included in the ESSDAI that are not appreciated in our study [2], which could potentially increase our cohort’s systemic involvement prevalence.

Overall, these findings are in accordance with those reported by the Sjögren Big Data Consortium [2] and the Italian GRISS cohort [8]. The only differences were the considerably higher frequencies of constitutional (28.2% *vs* 9.8% *vs* 12.6%) and cutaneous (23.6% *vs* 10.3% *vs* 13.1%) involvements in our cohort *vs* Big Data and GRISS, respectively. This may be due to a greater awareness and systematic clinical assessment in our more recent patient population. On the other hand, lymphadenopathic involvement was higher in our cohort compared with Big Data, but lower than in GRISS (15.7% *vs* 10.8% *vs* 24.5%), which may relate to different ethnogeographical backgrounds (higher percentage of Caucasian patients *vs* Big Data) or distinct protocols for lymphadenopathy assessment (e.g. regular CT/PET scans). Finally, it should be noted that articular involvement, distinct from the common, less-specific musculoskeletal pain that occurs in most SS patients, was indeed very frequent. This may be related to the assessment of these patients in a rheumatology department, with high awareness and expertise for these manifestations.

Of note, these results attest to the true systemic nature of SS. The notion of SS as a syndrome or nuisance limited to sicca, pain and fatigue should thus be abandoned, as previously proposed by Baer *et al.* [9], without neglecting the significance of the symptom burden that sicca, pain and fatigue mean for most SS patients. Furthermore, our findings are of particular interest when considering the risk of lymphoma and poor disease outcomes. Involvement in up to half of patients of the biological (hypergammaglobulinaemia, cryoglobulinaemia, low complement), glandular (parotid swelling), haematological (cytopenias), cutaneous (purpura) and lymphadenopathic (lymphadenopathies) domains reflects a high incidence of these risk factors [10].

We have identified phenotypes more likely to have active systemic disease. Younger and MSGB- and RF-positive patients are at an increased risk of systemic involvement. This is in agreement with previous knowledge of RF’s prognostic value in SS [4]. In addition, these features and positive ANA and/or anti-SSA were associated with a higher cumulative ESSDAI. This score has been proposed to add value in defining disease severity, by accounting for past organ involvement, which remains a sign of disease severity, even if currently absent [6]. As such, these patients should be closely monitored for extraglandular disease and long-term complications such as lymphoma. On the other hand, unlike previous reports [4], we could not demonstrate that anti-SSA per se is associated with systemic involvement. This may be due to the high prevalence of systemic involvement in our cohort, as detailed above. Autoantibody titres might possibly have provided additional prognostic information [11].

Our study also characterizes how systemic involvement varies throughout the disease course. When present, systemic involvement is usually found at diagnosis, but ≈40% of patients with no baseline systemic involvement will subsequently develop it. Furthermore, new/worsening domains are involved after baseline in 40–50% of patients, regardless of previous systemic disease. This is more likely to occur in patients with baseline constitutional involvement or RF. Additionally, although more than half of the patients have low baseline disease activity, >60% developed moderate or high disease activity later on (Fig. 1D). These findings regarding the natural history of SS have not, to the best of our knowledge, been previously reported and may assist in guiding clinical decisions and patient follow-up. Particular attention should be paid to incident systemic involvement in 40% of patients, as well as identifying the 60% of patients who will develop moderate–high disease activity and may require stronger immunosuppressants or be eligible for clinical trials. Prospective longitudinal studies are warranted to further acknowledge the fluctuations in disease activity and its associations with biomarkers and treatment. In the meantime, our work begins to uncover these aspects.

Limitations of our study include its retrospective nature, which may have decreased the accuracy of disease activity/severity characterization, with some patients having incomplete information (Supplementary Table S3, available at *Rheumatology Advances in Practice* online). Also, we did not have access to complementary measures such as EULAR SS Patient Report Index (ESSPRI) and salivary gland ultrasound. One additional limitation arises from the exclusion of patients meeting only the 2002 American-European Consensus Group criteria. While this constituted a minority of patients (3%), it has the potential to constrain meaningful comparisons with older studies. Adding to this, despite the comprehensive review of the assisting rheumatologists’ assessment, it remains plausible that certain medications could have contributed to some ESSDAI domains, such as immunosuppressant-induced cytopenias.

In conclusion, our study supports the concept of SS as a systemic complex disease, where the majority of patients have systemic involvement, including known risk factors for lymphoma. Younger and MSGB- and RF-positive patients are at higher risk of systemic disease and half of all patients will develop new organ involvement during follow-up. Close follow-up of these patients is warranted.

## Supplementary Material

rkae035_Supplementary_Data

## Data Availability

The original contributions presented in the study are included in the article. Further inquiries can be directed to the corresponding author.

## References

[1] Mariette X , Criswell LA. Primary Sjögren’s syndrome. N Engl J Med 2018;378:931–9. 29514034 10.1056/NEJMcp1702514

[2] Retamozo S , Acar-Denizli N, Rasmussen A et al Systemic manifestations of primary Sjögren’s syndrome out of the ESSDAI classification: prevalence and clinical relevance in a large international, multi-ethnic cohort of patients. Clin Exp Rheumatol 2019;37(Suppl 1):97–106. 31464664

[3] Romão VC , Talarico R, Scirè CA et al Sjögren’s syndrome: state of the art on clinical practice guidelines. RMD Open 2018;4:e000789. 30402274 10.1136/rmdopen-2018-000789 PMC6203093

[4] Brito-Zerón P , Acar-Denizli N, Ng W-F et al How immunological profile drives clinical phenotype of primary Sjögren’s syndrome at diagnosis: analysis of 10,500 patients (Sjögren Big Data Project). Clin Exp Rheumatol 2018;36(Suppl 1):102–12. 30156539

[5] Retamozo S , Brito-Zerón P, Ramos-Casals M. Prognostic markers of lymphoma development in primary Sjögren syndrome. Lupus 2019;28:923–36. 31215845 10.1177/0961203319857132

[6] Risselada AP , Kruize AA, Bijlsma JWJ. Clinical applicability of the EULAR Sjogren’s syndrome disease activity index: a cumulative ESSDAI score adds in describing disease severity. Ann Rheum Dis 2012;71:631. 22219139 10.1136/annrheumdis-2011-200766

[7] Ramos-Casals M , Solans R, Rosas J et al Primary Sjögren syndrome in Spain: clinical and immunologic expression in 1010 patients. Medicine (Baltimore) 2008;87:210–9. 18626304 10.1097/MD.0b013e318181e6af

[8] Quartuccio L , Baldini C, Bartoloni E et al Correlation between ESSDAI and ClinESSDAI in a real-life cohort of patients with Sjögren’s syndrome. Clin Exp Rheumatol 2017;35:546–7. 28229815

[9] Baer AN , Hammitt KM. Sjögren’s disease, not syndrome. Arthritis Rheumatol 2021;73:1347–8. 33559389 10.1002/art.41676

[10] Skarlis C , Argyriou E, Mavragani CP. Lymphoma in Sjögren’s syndrome: predictors and therapeutic options. Curr Treat Options Rheumatol 2020;6:1–17.

[11] Mariette X , Roux S, Zhang J et al The level of BLyS (BAFF) correlates with the titre of autoantibodies in human Sjögren’s syndrome. Ann Rheum Dis 2003;62:168–71. 12525388 10.1136/ard.62.2.168 PMC1754442

