# Supplementary material for: Beyond sicca: high prevalence and predictors of baseline and worsening systemic involvement in patients with Sjögren’s disease
Source: Rheumatol Adv Pract. 2024 Mar 6;8(2):rkae035. doi: 10.1093/rap/rkae035 (PMC10978570; doi:10.1093/rap/rkae035)
Supplement: rkae035_Supplementary_Data [file rkae035_supplementary_data.docx]

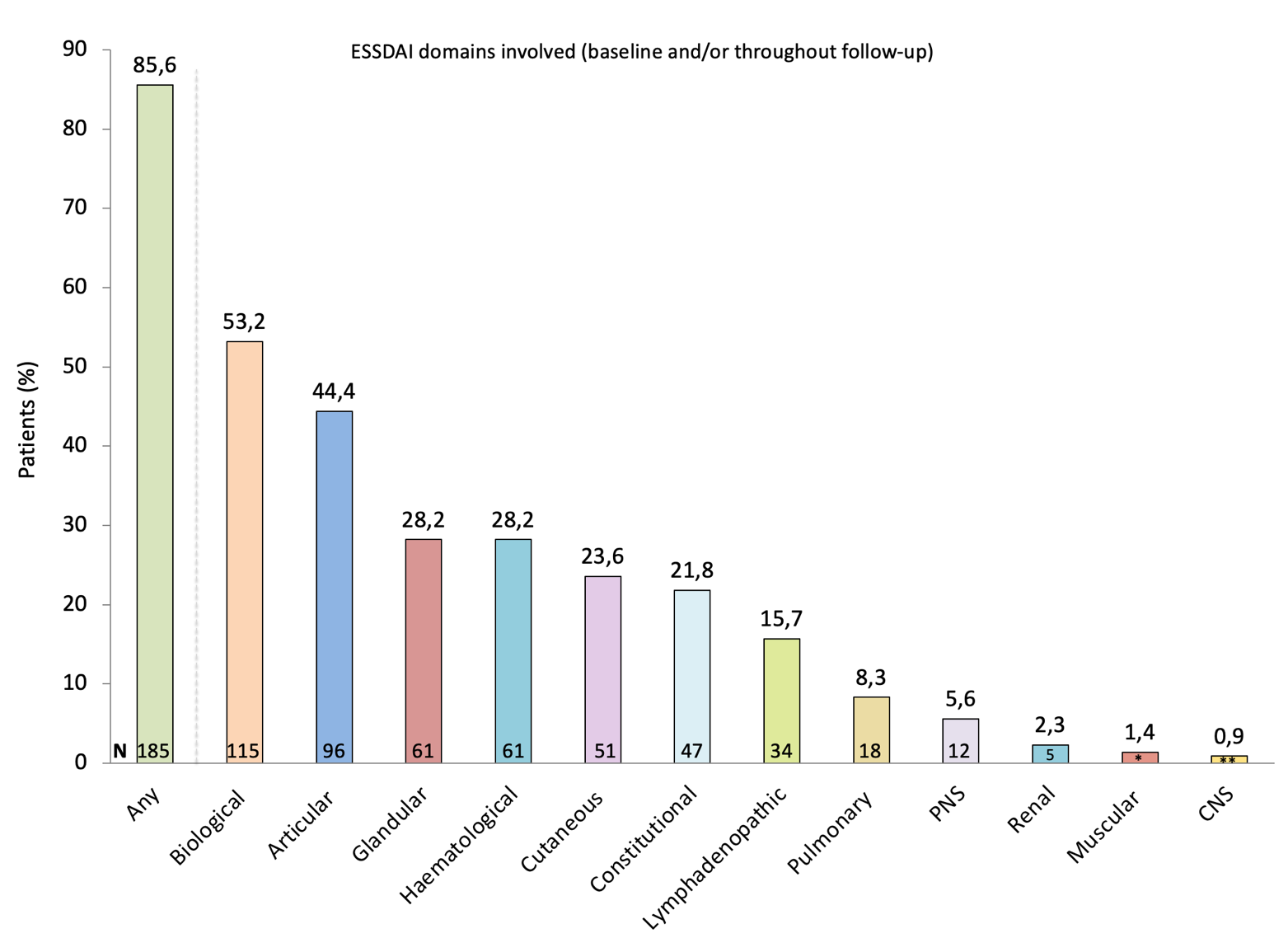
**Supplementary Figure S1 – Distribution of active ESSDAI domains throughout the entire course of the disease** (*3 patients; **2 patients)

**Supplementary Figure S2 -** Distribution of active ESSDAI domains in patients with systemic involvement at baseline and the ones developed during follow-up (*1 patient; **2 patients)


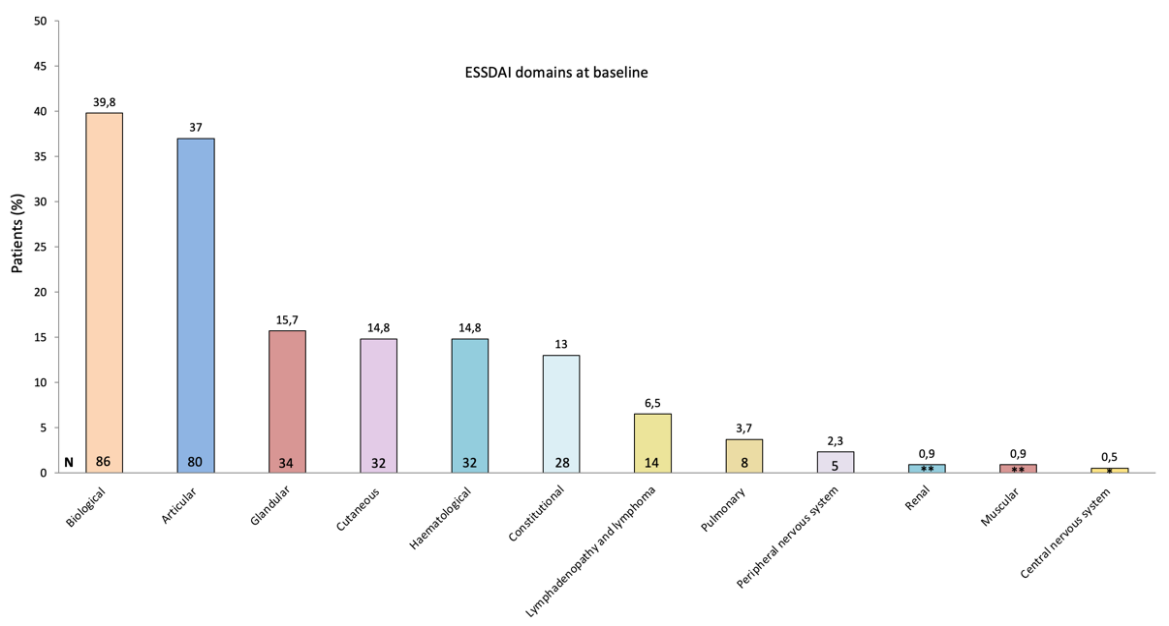

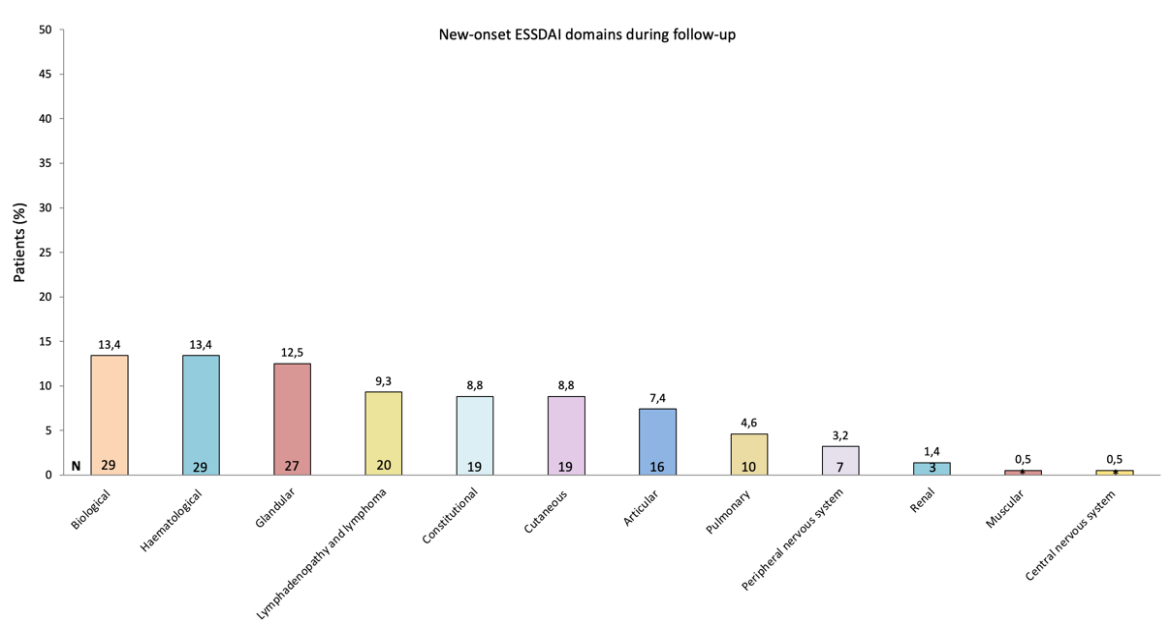


**Supplementary Table S1 –** Number of ESSDAI domains involved throughout the whole disease course.

| Number of ESSDAI domains | Patients (n) | Patients (%) |
| --- | --- | --- |
| 0 | 31 | 14.4 |
| 1 | 47 | 21.8 |
| 2 | 51 | 23.6 |
| 3 | 40 | 18.5 |
| 4 | 19 | 8.8 |
| 5 | 13 | 6.0 |
| 6 | 12 | 5.6 |
| 7 | 1 | 0.5 |
| 8 | 2 | 0.9 |

**Supplementary Table S2 –** Multivariate analysis for new and/or worsening ESSDAI throughout follow-up

|  | HR | 95%CI | p-value |
| --- | --- | --- | --- |
| Age | 1.01 | 0.99-1.02 | 0.407 |
| Sex | 0.69 | 0.17-2.84 | 0.607 |
| Anti-SSA | 2.41 | 0.84-6.95 | 0.102 |
| Anti-SSB | 0.94 | 0.59-1.50 | 0.791 |
| Positive minor salivary gland biopsy | 1.10 | 0.69-1.77 | 0.687 |
| **Rheumatoid factor** | **1.89** | **1.20-3.00** | **0.007** |
| Disease activity at diagnosis (low, moderate or high) | 0.73 | 0.48-1.12 | 0.149 |
| **Constitutional involvement at diagnosis** | **2.23** | **1.13-4.40** | **0.020** |

**Supplementary Table S3 –** Clinical and laboratorial features of patients in our cohort (with information on missing values)

|  | Overall  (n=216) | No systemic involvement (n=31) | Incident systemic involvement (n=22) | Systemic involvement at baseline (n=163) |
| --- | --- | --- | --- | --- |
| Age at diagnosis | 51.32±14.85 (216) | 58.16±13.90 (31) | 51.27±11.09 (22) | 50.02±15.18 (163) |
| Disease duration (in years) | 8.95±7.75 (216) | 5.90±5.78 (31) | 11.27±9.48 (22) | 9.22±7.70 (163) |
| Females | 209/216 (96.8) | 30/31 (96.8) | 21/22 (95.5) | 158/163 (96.9) |
| Ocular dryness | 198/216 (91.7) | 29/31 (93.5) | 21/22 (95.5) | 148/163 (90.8) |
| Oral dryness | 203/216 (94.0) | 30/31 (96.8) | 21/22 (95.5) | 152/163 (93.3) |
| Schirmer’s test < 5mm/5’ | 129/186 (69.4) | 19/26 (73.1) | 13/18 (72.2) | 97/142 (68.3) |
| Unstimulated salivary flow < 0.1mL/min | 52/121 (43.0) | 5/17 (29.4) | 3/11 (27.3) | 44/93 (47.3) |
| Positive minor salivar gland biopsy | 122/179 (68.2) | 12/27 (44.4) | 15/19 (78.9) | 95/133 (71.4) |
| Focus score | 2.54±1.48 (74) | 1.98±1.18 (8) | 2.56±1.66 (9) | 2.62±1.49 (57) |
| Anti-SSA/Ro | 198/216 (91.7) | 27/31 (87.1) | 21/22 (95.5) | 150/163 (92.0) |
| Anti-SSB/La | 117/215 (54.4) | 16/31 (51.6) | 13/21 (61.9) | 88/163 (54.0) |
| ANA | 201/216 (93.1) | 27/31 (87.1) | 20/22 (90.9) | 154/163 (94.5) |
| RF | 110/209 (52.6) | 7/28 (25.0) | 92/161 (57.1) | 11/20 (55.0) |
| Hematologic neoplasia | 11/216 (5.1) | 0/31 (0.0) | 1/22 (4.5) | 10/163 (6.1) |
| Cumulative ESSDAI | 8.35±8.07 (216) | 0.0±0.0 (31) | 7.59±7.37 (22) | 10.04±7.94 (163) |
| ESSDAI domains involved | 2.34±1.76 (216) | 0.0±0.0 (31) | 1.91±1.07 (22) | 2.84±1.63 (163) |
| Data shown as mean±SD (N) or n/N (%); Abbreviations: ESSDAI – EULAR Sjögren's syndrome disease activity index, SD – standard deviation, N – total number of patients with information of interest; n – number of patients positive for the variable of interest, ANA – antinuclear antibodies, RF – rheumatoid factor | | | | |
